# Morbidity and impact on quality of life in patients with indwelling ureteral stents: A 10-year clinical experience

**DOI:** 10.12669/pjms.313.6759

**Published:** 2015

**Authors:** Ioan Scarneciu, Sorin Lupu, Catalin Pricop, Camelia Scarneciu

**Affiliations:** 1 Ioan Scarneciu, MD, PhD, Clinic of Urology, Emergency Clinical County Hospital from Brasov, 25-27 CaleaBucuresti Street, 500326, Brasov, Romania. Faculty of Medicine, Transilvania University from Brasov, 29 Eroilor Boulevard, 500036, Brasov, Romania; 2 Sorin Lupu, MD, PhD, FECSM, Faculty of Psychology, SpiruHaret University from Brasov, 7 Turnului Street, 500152, Brasov, Romania. Clinic of Urology, Emergency Clinical County Hospital from Brasov, 25-27 CaleaBucuresti Street, 500326, Brasov, Romania; 3 Catalin Pricop, MD, PhD, Grigore. T. Popa University of Medicine and Pharmacy, 16 University Street, 700115, Iasi, Romania; 4 Camelia Scarneciu, MD, PhD, Faculty of Medicine, Transilvania University from Brasov, 29 Eroilor Boulevard, 500036, Brasov, Romania

**Keywords:** Ureteral stents, Complications, Tolerability, Quality of life

## Abstract

**Objective:**

Prospective analysis of the prevalence of symptoms, tolerability and complications associated with ureteral stents and their impact on quality of life based on the Flanagan Quality of Life Scale and a not-validated questionnaire from our clinic.

**Methods:**

A total of 2200 adult patient participated to this study in a period of 10 years (2003-2012). Those patients were asked to complete the QOLS and a not-validated questionnaire from our clinic, before ureteral indwelling, 7 day after ureteral indwelling and 14 days after removal of the stent.

**Results:**

Total 1520 patient aged between 18 and 84 years completed the study. The analysis of data showed that the unpleasant symptoms caused by stent were encountered more frequently at 7 days after stent insertion, in terms of urinary frequency, dysuria, urgency and macroscopic haematuria, this difference being statistically significant (p<0.05). After analysis the responses to QOLS questionnaire, at 7 days after stent placement, mean scores show a clear reduction in the QoL of those patients, in all cases the standard deviation being at a great value, indicating a high variability of responses, but at 14 days after its suppression of stent the average scores are somewhat closer to the baseline.

**Conclusions:**

Our study brings many elements that shows a statistically significant increase in the incidence of numerous side effects and impaired quality of life. It contributes to existing data from the literature as regards the knowledge of the pathology determined by the presence of foreign body in the urinary tract and in providing patient counseling.

## INTRODUCTION

The double J ureteral stent (JJ stent) is a catheter which is placed in to the ureter lumen in order to maintain its permeability. The indwelling of the double J ureteral stents (DJS) represents a very frequently used method within the urological practice, in order to ensure the drainage of the urine from the superior urinary tract.^1^ Since the first description within urological practice of DJS and since the first use of DJS (Finney and Hepperlen in 1978), those have suffered changes with regard to the structure of the material and their shape.^2^ Recently the use of stents with metallic inserts has been suggested, also stents covered by special gels or bioactive substances, such as antibiotics, anti-fungicides or heparin. The use of biodegradable materials within the structure of stents has also been taken into account, in order to avoid repeating endoscopic mounting procedures, as well as suppression of stents^3^,^4^ but the ideal stent, which should be supposed to offer optimal urinary drainage, long term efficiency and maximum tolerability for the patient, is not yet available.^2^,^3^,^5^,^6^

## METHODS

The study is based on prospective analysis of 2200 cases within which DJS have been placed, all operations being performed within the Clinic of Urology, Emergency Clinical County Hospital from Brasov, Romania, from 2003 to 2012.

Patients who required DJS placement were aged between 18 and 84 years. In all cases the informed consent was obtained before performing any urological procedures and all ethical procedures and protocols from our hospital were respected. The way of placing DJS was generally retrograde using the cystoscope or subsequent to performing retrograde ureteroscopy. In some cases the stent placement has been made in open surgery. We always tried to maintain the stent over a small period of time, considering possible complications of the internal urinary drainage. Within the protocol we included Flanagan Quality of Life Scale (QOLS) (16 items) and a not-validated questionnaire developed by the team members, which followed the concentration of the parameters with urologic significance that must be followed, in terms of the presence and degree of severity. Within this questionnaire varying degrees of the following clinical parameters were determined: urinary frequency, dysuria, urgency, suprapubic pain, radiating lumbar pain, macroscopic haematuria. Signs and symptoms were ranked from 1 (no or minimal) to 5 (maximum intensity). The questionnaires were given to patients in three distinct moments of time: before DJS placement, 7 days from DJS placement and 14 days after the DJS removal, and the results were analyzed according to the composition material of every stent used. In QOLS the scores range is between 16 and 112 (average for healthy population is 90). High scores reflect an enhanced quality of life.^7^

In evaluation of the quality of life (QoL) for these patients we didn’t use Ureteral Stent Symptom Questionnaire validated by Joshi et al. in March 2003^2^,^8^ because at the start of our study this was not available. For correct analysis of results we take into consideration only the cases were we could apply the protocol that we have proposed (1520 patients). All patients who were included in the study were followed prospectively and for statistical analyses we used SPSS soft.

## RESULTS

Out of 2.200 patients with indwelled ureteral stent, 61.63% (n=1356) were males and 38.36% (n=844) were females. Stent maintenance period was between 5 and 218 days, with an average of 31 days.

We used stents 6-7 Ch stents, with a length of 24-28cm. Distribution of cases according to the composition of stent was: aliphatic polyurethane (40.98%), hydrophilic polyurethane coating (20.72%), carbothane (17.82%), silicon (20.46%). The choice of stent was random, depending on stents available in our clinic at that time, with exceptions were patients who required long time internal urinary drainage for whom the use of “long-life” stents (carbothane) was taken into account. In Tables-II, III and IV we present results of all data.

**Table-I:** Distribution of cases according to indication of ureteral stent mounting

Placement indication	No. of patients	Percentage
Obstructive anuria	264	12%
After ureteroscopy	748	34%
Push-back of superior ureteral stones	176	8%
DJS in open surgery procedures:		
- Pyeloplasty	132	6%
- Pielolitotomy	154	7%
- Ureterolithotomy	44	2%
Oncologic diseases	418	19%
Before performing ESWL	66	3%
Emergency internal urinary drainage	198	9%

**Table-II:** Results obtained after applying of our not-validated questionnaire.

	Before stent indwelling				At 7 days after the indwelling of stent				After removal of the stent (14 days)			
	A	B	C	D	A	B	C	D	A	B	C	D
Urinary frequency	0.48%	0.95%	1.47%	0.64%	63,24%*	53.65%*	52.02%*	46.62%*	10.91%	5.71%	6.27%	4.82%
Dysuria	2.24%	3.17%	2.21%	2.89%	59.55%*	57.46%*	44.64%*	55.94%*	21.82%	9.2%	7.01%	6.75%
Suprapubic pain	5.77%	8.88%	5.16%	9.32%	30.17%	30.47%	33.94%	36.65%	3.85%	1.26%	2.21%	1.92%
Urgency	1.92%	1.9%	2.95%	2.89%	60.35%*	44.12%*	46.49%*	45.98%*	10.27%	8.57%	6.27%	7.71%
Lumbar pain	13.8%	18.4%	18.81%	15.75%	20.06%	24.44%	26.19%	24.75%	1.12%	0.95%	1.1%	2.57%
Macroscopic haematuria	1.92%	2.22%	3.32%	2.25%	64.68%*	51.42%*	54.98%*	45.98%*	5.77%	4.76%	4.05%	3.85%
Persistent macroscopic haematuria	1.28%	1.58%	5.16%	1.92%	29.53%*	23.8%*	23.61%	18%*	1.44%	0.63%	0.73%	0.96%

*Legend:* A – aliphatic polyurethane; B – hydrophilic polyurethane coating; C – carbothane; D – silicone;

* p<0.05

**Table-III:** Results obtained from QOLS.

	Before indwelling stent		At 7 days after the indwelling of stent		After removal of the stent (14 days)	
	Mean (average)	Standard deviation	Mean (average)	Standard deviation	Mean (average)	Standard deviation
Aliphatic polyurethane (n=623)	88,74	19,24	68,03	22,83	81,3	21,32
Hydrophilic polyurethane coating (n=315)	88,24	16,85	69,13	20,9	81,04	16,9
Carbothane (n=271)	62,89	14,65	59,67	16,79	64,33	18,93
Silicone (n=311)	86,98	16,73	79,67	14,34	86,32	20,3

**Table-IV:** Distribution of cases by the complications after indwelling the DJS.

Complication	Percentage	Comments
Urinary tract infection	9,01% (n=137)	no severe
Fever	6.11% (n=93)	evolution favorable
Malposition	(0.98%) (n=15)	solved by removing stent
Superior or inferior ureteral migration	4.01% (n=61)	no
Inadequate relief of obstruction	20.72% (n=315)	17.82% stent replacement was required
Encrustation (See Fig. 1, 2 and 3)	15% (n=228)	4 cases (0,92%) – ESWL 6 cases (0,39%) ureteroscopy or cystolitholapaxy
Stent fracture	1,11% (n=17)	removal of stent fragments
Ureteral erosion or fistulization	no	no
Forgotten stent	0,19% (n=3)	no
Stenturia	no	no

**Fig.1:**
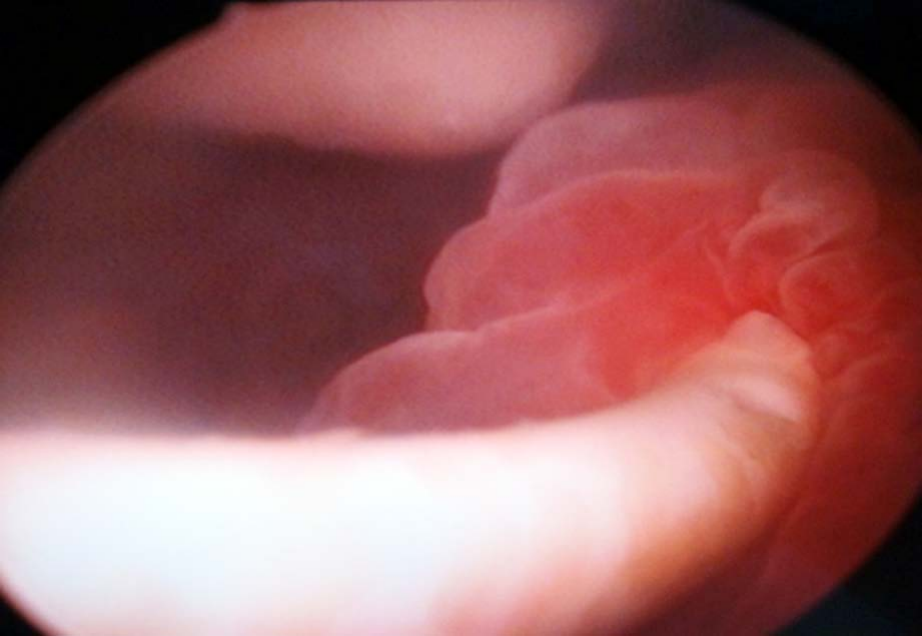
Edematous lesion of ureteral meatus, caused by ureteral stent (cystoscopic aspect).

**Fig.2:**
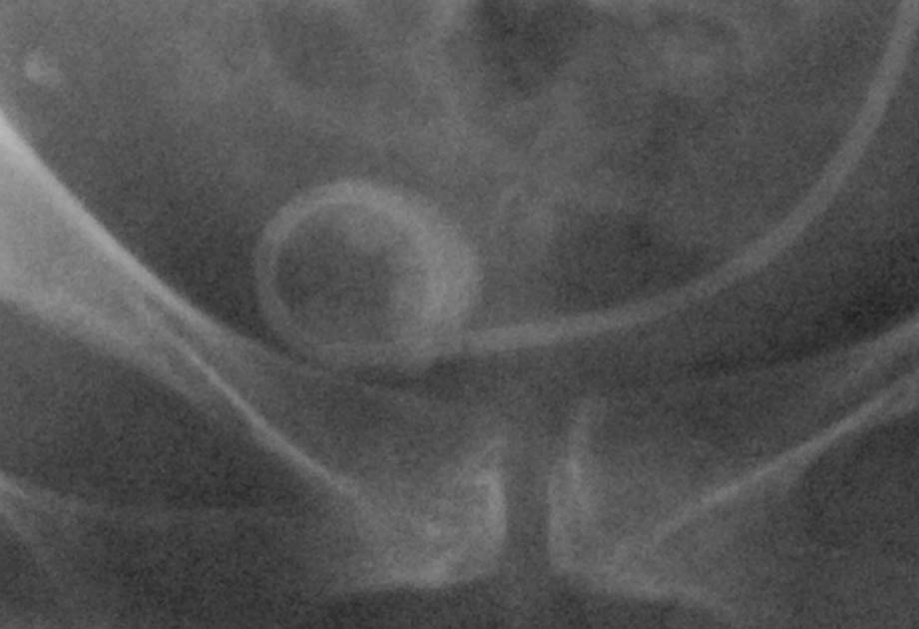
Stent encrustration of bladder coil (radiography aspect).

**Fig.3:**
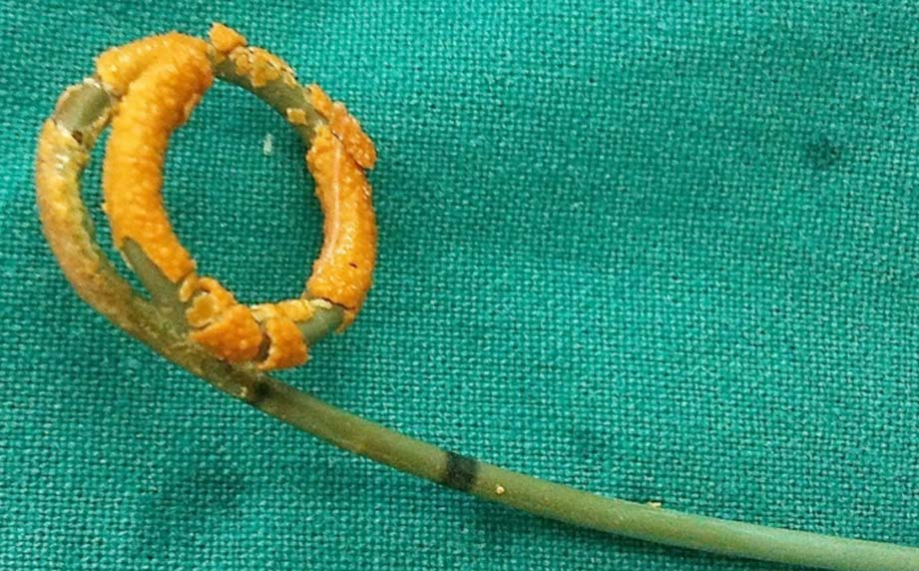
Stent encrustration of proximal coil (aspect after stent removal).

## DISCUSSIONS

In this 10-year prospective study, it is evident that the presence of DJS cause varying degrees of discomfort to patients. Analysis of data revealed differences but statistically insignificant between the 4 types of stent used. None of the materials proved to be superior in terms of secondary manifestations of this foreign body in the urinary tract.

Urinary frequency and urgency are symptoms directly caused by mechanical factor.^2^ The majority of patients complain of these symptoms increased significantly during the day, highlighting the dependency of physical activity. Bladder muscle over activity is clearly enhanced by the presence of DJS. The urinary frequency and urgency were present in statistical significant percentage 7 days after the stent placement (p <0.05).

Dysuria seems to be more common when using stents with excessive length, in this study dysuria appeared in statistically significant proportion (p<0.05) at 7 days after stent installation and remain after its removal, but without statistical significance in this case. Suprapubic pain is caused by direct irritation of the bladder mucosa determined by stent, but can be exacerbated in the case of secondary infection or stones in the distal volute.^2^,^9^ In our study this is obviously more common after mounting the stent, but statistically insignificant in this group, and relatively very similar percentage of the base value 14 days after stent removal.

Back pain is caused by vesicoureteral reflux, being secondary to the temporary cancellation of the intramural antireflux mechanism.^2^,^9^ We observed a clear increase but not statistically significant incidence of back pain.

Haematuria is a very common sign, being dependent mainly by physical activity, by mucosal microtrauma. Single episode or intermittent haematuria was present in statistically significant percentage of patients with DJS and persisted at 14 days after stent removal (but not significant). Persistent haematuria highlighted a statistically significant increase in patients with DJS, after suppressing of internal drainage the percentage approaching clear to the base value.

The results are largely consistent with the literature, many authors noting such side effects that persist throughout all the stenting time. Ilkram Ullah et al. showed urinary frequency and urgency in 68% of cases, dysuria in 70% of patients, haematuria in 53.4% and lumbar pain in at least one third of cases.^11^ Chew BH et al.^9^, Haleblian G et al.^12^, Sur RL et al.^13^, Lingeman JE et al.^14^, Leibovici D et al.^15^ show frequency, urgency and dysuria in 50-60% of cases, low back pain in 19-32 % of cases, suprapubic pain in 30% and haematuria in 25% of patients. Joshi et al., using the validated questionnaire USSQ, revealed that in approximately 80% of cases the stent caused a degree of pain that affected the good functioning of daily activities, including work capacity.^8^,^16^ There are authors who have shown different results: Damiano et al. reported irritative symptoms in 37% of patients and macroscopic haematuria in 18%, far less frequently than those highlighted from the analysis of data in our group.^11^,^17^

As regards stent complications like urinary tract infection, encrustration, migration, spontaneous fracture, malposition, inadequate relief of obstruction or forgotten stent, our results are relatively similar to those in the literature. Regarding the results of QOLS, mean scores before the stent was used were relative similar, close to 90, except cases were a carbothane stent was used (majority with cancer), in which cases the quality of life is profoundly affected due to disease itself. At 7 days after stent placement, mean scores show a clear reduction in the QoL for those patients, but at 14 days after its suppression the average scores are somewhat closer to the baseline. In all cases the standard deviation was at a great value, indicating a high variability of responses, reflecting the different ability of patients to cope with distressing symptoms caused by this foreign body, strengthening more once the idea that “there are no diseases, but sick people”. Our results further contribute to studies of other authors. Thus, during stenting period of time Leibovici D et al. show a significant percentage of sleep disorders, anxiety, decreased of libido and other sexual dysfunction, 45% of patients reporting reduced quality of life^15^ and Joshi demonstrated a reduction by 80% of quality of life in patients with DJS.^8^,^16^

Modern science still offers many alternatives in order to invent the “ideal stent”. Thermo-expandable stents are increasingly studied, thermo-expandable shape memory stents, stents made of biodegradable or bioabsorbable materials, coated stents with various substances as heparin, various enzymes, hydrogel, antibiotics and antifungal medication or anti-inflammatory medication.^3^,^9^,^12^,^18^-^20^

## CONCLUSIONS

Although it is a real success of modern technology and the element that in many cases help us to save the kidney, DJS may cause some side effects and impaired quality of life of patients that are not neglected. Our study, that was conducted on a large number of patients, followed prospectively, bring many elements that shows a statistically significant increase in the incidence of numerous side effects and impaired QoL, further contributing to existing data from the literature as regards the knowledge of the pathology determined by the presence of foreign body in the urinary tract. Although most of the complications caused by the stent does not threaten the patient’s life, it is the duty of those involved in the care of patients to bring more information and results of their experiences and contribute to finding innovative solutions, to some of increasingly effective biomaterials, because the use of ureteral stents, even if they are not the “ideal”, is indispensable in modern urology.
